# A Novel Bi-Directional Channel for Nutrient Uptake across Mycobacterial Outer Envelope

**DOI:** 10.3390/microorganisms12091827

**Published:** 2024-09-04

**Authors:** Lei Liu, Chongzheng Wen, Xiaoying Cai, Weimin Gong

**Affiliations:** Division of Life Sciences and Medicine, Hefei National Research Center for Interdisciplinary Sciences at the Microscale, University of Science and Technology of China, Hefei 230026, China; lycan@mail.ustc.edu.cn (L.L.); czwen@mail.ustc.edu.cn (C.W.); caixy@mail.ustc.edu.cn (X.C.)

**Keywords:** mycobacterial outer envelope, TiME, nutrient uptake, survival at acid pH, antibiotics

## Abstract

Nutrients are absorbed by special transport proteins on the cell membrane; however, there is less information regarding transporters across the mycobacterial outer envelope, which comprises dense and intricate structures. In this study, we focus on the model organism *Mycolicibacterium smegmatis*, which has a cell envelope similar to that of *Mycobacterium tuberculosis,* as well as on the TiME protein secretion tube across the mycobacterial outer envelope. We present transcriptome results and analyze the protein compositions of a mycobacterial surface envelope, determining that more transporters and porins are induced to complement the deletion of the *time* gene in *Mycolicibacterium smegmatis*. The TiME protein is essential for nutrient utilization, as demonstrated in the uptake experiments and growth on various monosaccharides or with amino acids as the sole carbon source. Its deletion caused bacteria to be more sensitive to anti-TB drugs and to show a growth defect at an acid pH level, indicating that TiME promotes the survival of *M. smegmatis* in antibiotic-containing and acidic environments. These results suggest that TiME tubes facilitate bi-directional processes for both protein secretion and nutrient uptake across the mycobacterial outer envelope.

## 1. Introduction

Tuberculosis (TB), caused by the *Mycobacterium tuberculosis* (Mtb) complex, stands as one of the recorded devastating plagues in human history [1,2]. Landmark advancements in combating TB, including the development of the BCG (Bacillus Calmette-Guérin) vaccine and the discovery of streptomycin, significantly shield humanity from its ravages [1]. However, drug-resistant TB is becoming a significant public health challenge in many regions worldwide [3,4]. 

The extremely complex cell envelope and immune escape strategies of Mtb empower it to endure harsh environments, protecting the bacteria from annihilation [5,6,7]. The multi-layered mycobacterial envelope consists of cytoplasmic membrane, a cell wall (~14 nm) composed of peptidoglycan covalently linked to arabinogalactan and anchored in mycomembrane-containing mycolic acids ensconced by glycolipids [8,9], and a capsule (~35 nm) made up of proteins, polysaccharides, and lipids on the cell surface [10,11]. Consequently, the mycobacterial envelope exhibits low permeability, necessitating porins to breach this barrier and facilitate transport into and out of cells [12,13,14]. *Mycolicibacterium smegmatis* is a non-pathogenic bacterium that shares similarities in its cell envelope with Mtb and is often used as a model organism for studying pathogenic mycobacteria [15]. 

MspA (MSMEG_0965), the initial reported mycomembrane porin from *M. smegmatis*, is a homooctameric goblet-like channel (Supplementary Figure S1a) [13,16] that mediates nutrient uptake and drug diffusion through the mycomembrane [17,18]. However, there exists no orthologous protein of MspA in Mtb. Recent studies have highlighted various transport-related proteins located on the outer envelope of Mtb. These include PE/PPE proteins, facilitating nutrient transport [19,20,21,22], and the EsxE-EsxF complex involved in toxin secretion [23]. Despite suggestions of some PE/PPE proteins possessing pore-forming capabilities [20,24,25], direct structural evidence confirming their role as porins is lacking. Therefore, the nutrient channels in the outer envelope of Mtb remain an unsolved mystery.

TiME (Rv3705c/mtTiME in Mtb and MSMEG_6251/msTiME from *M. smegmatis*) represents a highly conserved protein within the *Mycobacteriaceae* family, serving as the first identified protein transport tube across the mycobacterial outer envelope [14]. TiME is highly conserved in the gene loci, sequence, and structure between Mtb and *M. smegmatis*. The N-terminal segment of the full-length TiME protein is highly hydrophobic and is predicted to be a general secretion (Sec)-specific signal peptide and forms a transmembrane helix (N-terminal helix α0) [14]. TiME assembles into two layers of rotationally symmetric rings, boasting an inner diameter of 50~60Å, large enough for the passage of molecules (Supplementary Figure S1b) [14]. However, only the full-length TiME protein, containing the N-terminal helix α0, forms a transmembrane pore on the lipid bilayer [14]. These ring-shaped complexes align in a tail-to-tail configuration, collectively shaping outer envelope tubes confirmed through cryo-electron microscopy (cryo-EM) and subcellular localization [14,26,27]. In the absence of TiME, a considerable reduction in secreted proteins occurred despite the normal levels of these protein mRNAs in the *M. smegmatis*. However, this effect diminished when bacteria were cultured with Tween 80, which disrupts the capsule and cell wall surface [14].

In this study, we conducted mRNA-seq analysis on both wild-type and Δ*mstime M. smegmatis* strains, revealing the impact of TiME depletion on hundreds of genes and notably altering transporter activity in *M. smegmatis*. Moreover, our findings confirm TiME’s role in facilitating nutrient uptake and utilization, which is essential for the survival and optimal growth of *M. smegmatis*. Our results indicate that TiME forms a distinctive bi-directional channel across the mycobacterial outer envelope, enabling nutrient uptake besides protein secretion.

## 2. Materials and Methods

### 2.1. Mycolicibacterium Smegmatis Strains and Clone Construction

*M. smegmatis* mc^2^155 *mstime* (*msmeg_6251*gene) knock-out strain (Δ*mstime*) was constructed by allelic recombination, which was described in a previous report [14]. Briefly, the pJV53-GFP plasmid was electrotransformed into *M. smegmatis* mc^2^155 to generate competent *M. smegmatis* cells containing the gp60 and gp61 proteins of mycobacteriophage Che9c [28,29]. The PUC-Hyg-P1-P2 plasmid, which contains DNA fragments upstream (P1) and downstream (P2) of the *mstime* gene, was then electrotransformed into these competent cells to facilitate recombination with the *mstime* gene [14]. The transformed cells were incubated at 37 °C for 4 h to recover and were then plated on Middlebrook 7H10 agar (BD Difco^TM^) with supplements. After 3 days of incubation, colonies were selected for verification by colony PCR. Successfully recombined colonies were further cultured, and pJV53-GFP was eliminated by growing them on 7H10 agar with 10% sucrose. GFP-negative colonies were PCR-verified and sequenced. A Western blot confirmed that the *mstime* gene was not expressed in the knock-out strain [14]. 

Four strains (Δ*mstime*::*mstime*, Δ*mstime*::*mstime*^G33R-G50R-A91R^ mutant, Δ*mstime*::*mttime*, and Δ*mstime*::*mttime*^G31R-G48R-A89R^ mutant) with msTiME or mtTiME constitutive expression were used here (Supplementary Table S1). Respectively, the *mstime*/*mttime* (*rv3705c*) gene and *mstime*^G33R-G50R-A91R^/*mttime*^G33R-G50R-A91R^ mutant gene were cloned into the pMV361D vector derived from the pMV361 plasmid [14] between *EcoR* I and *Hind* III sites. Related primers and plasmids information were listed in Supplementary Table S1 and S2. Plasmids were transformed into *M. smegmatis* mc^2^155 Δ*mstime* strains to recombine the gene on the plasmid to the attachment site. 

### 2.2. Bacteria Culture Conditions

*M. smegmatis* monoclonal strains were grown in 20 mL of 7H9 (BD Difco) broth A (Supplementary Table S3) medium with 0.02% (*w*/*v*) Tyloxapol at 37 °C for 2 days. A total of 50 μg/mL kanamycin was added to cultivate the bacteria carrying pMV361D plasmids. 

For the growth assay on different monosaccharides, the saturated bacteria were subcultured with an initial OD_600nm_ of 0.005 by inoculating into 50 mL of fresh 7H9 broth B (Supplementary Table S3) medium without antibiotics. Glucose, fructose, and xylose were added before culturing as the only carbon source in each medium, respectively. For the concentration determination, the bacteria solution with an OD_600nm_ > 0.8 was diluted five times by the 7H9 broth B medium with 0.02% (*w*/*v*) Tyloxapol. The bacteria cultured without 0.02% (*w*/*v*) Tyloxapol were collected and mixed with 7H9 broth B containing 0.1%~1% (*w*/*v*) Tyloxapol to disperse before measuring the OD_600nm_. Growth rates were determined in three independent cultures by the OD_600nm_ measurements every 6 h.

For the growth assay on different amino acids, *M. smegmatis* strains were grown in the 7H9 broth A medium with 0.02% (*w*/*v*) Tyloxapol at 37 °C to an OD_600nm_ of approximately 0.8. Two to four OD_600nm_ of bacterial cell cultures were collected and washed by minimal Sauton’s medium without amino acids (Supplementary Table S3) and then suspended. The bacteria solution was static for 5 min to obtain the upper suspensions and remove aggregate bacterial particles. The suspensions were diluted to an OD_600nm_ of 0.2 with the medium. These bacteria were further diluted in the different amino acid medium with a 1:1000 dilution. The assay was performed in 96-well black clear-bottom plates at 100 μL/well, and the minimal Sauton’s medium was also added as a blank control. After 2 days of incubation at 37 °C, Alamar Blue solution (ThermoFisher, Waltham, MA, USA) at 10 μL/well was added with the fluorescence recorded after another 24 h incubation at 37 °C with an excitation at 540 nm and emission at 590 nm by a microplate reader (CLARIOstar^®^, BMG LABTECH, Ortenberg, Germany).

For the growth assay at an acidic pH, the saturated bacteria were subcultured by inoculating into a 50 mL defined minimal medium, of which the sole carbon source was glycerol or glucose (Supplementary Table S3) with an initial OD_600nm_ of 0.01. Strains without capsules were cultured by adding 0.02% (*w*/*v*) Tyloxapol in the medium. The growth rates were determined in three independent cultures by OD_600_ measurements every 6 h.

### 2.3. Transcriptome Sequencing

Transcriptome analyses were conducted with both the wild-type and Δ*mstime* samples of the *M. smegmatis* mc^2^155 strain at the exponential phase (OD_600nm_ ~1.0). The transcriptome of *M. smegmatis* was sequenced and analyzed by Sangon Biotech (Shanghai) Co., Ltd. (China). Part of the transcriptome sequencing data was used to analyze the role of msTiME in protein secretion. The mRNA level for 74 secreted proteins, detected by quantitative mass spectrometry, along with 56 components of known secretion systems in *M. smegmatis’* plasma membrane, was listed, as described in detail in a previous report [14]. The differential expression analyses of two samples were conducted by employing DESeq2. GO enrichment analysis was performed using TopGO, while ClusterProfiler was utilized for the classification and enrichment analysis of the KEGG (Kyoto Encyclopedia of Genes and Genomes) pathways and COG (Clusters of Orthologous Groups). 

### 2.4. Protein Extraction from Outer Envelope of M. smegmatis

To extract the proteins from the outer envelope, wild-type *M. smegmatis* mc^2^155 were grown in the 7H9 broth A medium at 37 °C to an exponential phase and then harvested by centrifugation at 3000× *g* for 15 min at 4 °C. Bacteria were washed softly with phosphate-buffered saline (PBS) buffer (1.47 mM KH_2_PO_4_, 8.1 mM Na_2_HPO_4_, 2.67 mM KCl, and 138 mM NaCl, pH 7.4). Then, the bacteria were incubated with PBS buffer containing 1% Tween 80 for 30 min at room temperature to separate the surface layer fraction. The resulting supernatant by centrifugation was filtrated with a 0.22 μm pore size filter to remove residual cells. The filtrate was treated with precooled TCA to a final concentration of 10% (*v*/*v*); the mixture was incubated on ice for 1 h and then centrifuged at 36,800× *g* for 15 min at 4 °C. The precipitate was washed with pre-cold acetone three times. The supernatant was discarded very carefully, and the protein pellet was air-dried and weighed. The appropriate protein pellet was resuspended in 2× loading buffer prepared for SDS-PAGE.

### 2.5. Saccharide Uptake Assay

*M. smegmatis* monoclonal strains were grown as before. The 2 mL cultures were diluted in 200 mL of fresh 7H9 broth A medium and subcultured at 37 °C for 48 h to obtain *M. smegmatis* with a capsule. Cells without capsules were subcultured in the 7H9 broth A medium, adding 0.02% (*w*/*v*) Tyloxapol for 20 h. Bacteria were harvested by centrifugation at 8000× *g* for 30 min at 4 °C and resuspended in PBS buffer with a concentration of approximately 3 mg dry weight mL^−1^ after being washed twice. NBD-labeled monosaccharides (Supplementary Table S4) were added to 500 μL of bacteria suspensions at final concentrations of 50 μg/mL. As a control, same-volume PBS buffers were added to 500 μL of the bacteria suspensions to obtain background fluorescence intensities. The mixtures were incubated at 25 °C with shaking at 200 rpm for 2 h; then, bacteria were collected by centrifugation at 16,200× *g* for 10 min and washed once with 850 μL of PBS buffer. Finally, the bacteria were resuspended in 100 μL of PBS buffer, and fluorescence intensities of the NBD accumulation in cells were detected at an excitation wavelength of 485 nm and an emission wavelength of 535 nm by microplate reader.

### 2.6. Anti-TB Drug Sensitivity Experiments

Susceptibilities of different *M. smegmatis* strains to rifampicin and streptomycin were tested by a gradient broth dilution method [20]. The strains were grown and collected and then diluted to an OD_600nm_ of 0.2 with the 7H9 broth A medium, the same as the growth assay on different amino acids. The bacteria (with a further 1:1000 dilution) in different concentrations of rifampicin or streptomycin were added to 96-well black clear-bottom plates at 100 μL/well and grown at 37 °C for 24 h. Alamar Blue solution (ThermoFisher) at 10 μL/well was added with a 15 h incubation at 37 °C, and the fluorescence was recorded with the excitation at 540 nm and emission at 590 nm by a microplate reader (SpectraMax iD5, Molecular Devices, San Jose, CA, USA).

## 3. Results

### 3.1. Transcriptome Analysis of Wild-Type and Δmstime M. smegmatis Strains

In a previous study, we generated a *mstime* (*msmeg_6251* gene) knock-out strain of *M. smegmatis* via allelic recombination and confirmed that msTiME facilitated protein secretion across the outer cell envelope [14]. RNA-seq analysis was conducted to explore the differences between wild-type and Δ*mstime M. smegmatis*. Of the 6770 sequenced genes, 869 genes exhibited significant changes (>2-fold change with an adjusted *p*-value of less than 0.05) at the transcriptional level (Supplementary Data S1). Notably, despite a marked decrease in secreted proteins within the Δ*mstime* culture medium, most detected secreted proteins and known secretion system components showed no significant changes at the mRNA level post-mstime deletion, as detailed in the prior report [14].

**Figure 1:**
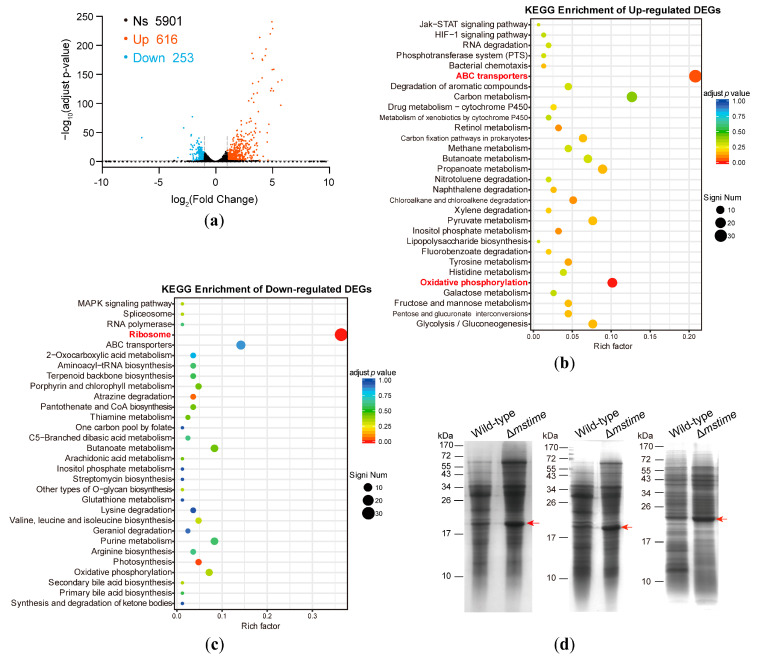
Transcriptome analysis and comparison of outer envelope protein composition between wild-type and Δ*mstime M. smegmatis* strains. (**a**) Volcano plot displaying the gene expression profiles of wild-type and Δ*mstime*. Axes represent the gene expression values. |log2 Fold Change| > 1 and adjusted *p*-values < 0.05 are noted as DEGs (differentially expressed genes). The red and blue dotted lines delineate the 2-fold cut-off in gene expression levels between the two compared samples, revealing that the majority of genes fall within these boundaries. Red dots signify upregulated genes (Up); dots in blue are downregulated genes (Down); those in black indicate no significant change (Ns). (**b**,**c**) Enrichment analysis of the KEGG pathway for annotated DEGs.The top 30 enriched functions are classified and presented. The ‘Rich factor’ means the ratio of DEGs to all genes within a particular functional category. Dot size correlates with the number of DEGs in the pathway, while dot color reflects the adjusted *p*-value. ‘Signi Num’ indicates significant gene numbers. (**d**) Outer envelope protein composition. SDS-PAGE-based analysis of outer envelope protein in wild-type and Δ*mstime* cultured in the medium without Tyloxapol. The red arrows denote a notably distinct protein band between wild-type and Δ*mstime*. The experiment was conducted with at least three independent biological replicates.

Comparative analyses between the wild-type strain and Δ*mstime* revealed 616 upregulated genes and 253 downregulated genes (Figure 1a and Supplementary Data S1). These differentially expressed genes (DEGs) underwent Gene Ontology (GO) classification, highlighting their involvement in diverse biological processes, primarily enriched in transporter activity (Supplementary Figure S2a). Additionally, the KEGG (Kyoto Encyclopedia of Genes and Genomes) pathway analysis demonstrated the varied impacts of DEGs on metabolic pathways. Specifically, the enrichment of upregulated DEGs mainly focused on ABC transporter and oxidative phosphorylation pathways, while downregulated DEGs were centered on ribosomal pathways (Figure 1b,c). The COG (clusters of orthologous groups of proteins) analysis corroborated these observations, highlighting the noteworthy enrichment in carbohydrate and amino acid transport and metabolism (Supplementary Figure S2b,c). Among the 616 upregulated genes in the Δ*mstime* strain, 73 genes were referred to carbohydrate transport and metabolism, and 42 genes were involved in amino acid transport and metabolism (Supplementary Data S1). These prominent enrichments in scatter plots suggest a potential association of msTiME with nutrient transportation. Furthermore, upregulated DEGs related to energy production and conversion could potentially fuel transportation metabolism (Figure 1b). Thus, we posit that the msTiME channel may play a role in nutrient uptake in addition to its role in secretory functions.

### 3.2. TiME Deletion Triggers Elevated Expression of MspB or Its Paralogues 

Since msTiME predominantly distributes in the cell wall and capsule of *M. smegmatis* [14,26], we extended our analysis to investigate the protein compositions within the outer envelope of both wild-type and Δ*mstime* strains using SDS–polyacrylamide gel electrophoresis (SDS-PAGE). The results revealed a notably intensified band in Δ*mstim* at a molecular weight ranging between 17 and 26 kDa (highlighted by the red arrow in Figure 1d). Subsequently, the band was digested, and the resulting peptides were identified as porin MspB (MSMEG_0520) by mass spectrometry (Supplementary Data S2). However, considering that MspA has three paralogous proteins (MspB, MspC, and MspD) exhibiting a marginal difference in their amino acid sequences [18], the identified peptides (Supplementary Data S2) could not be precisely attributed to MspA-D. Our observations suggest that the transcriptions of MspA-D were variably increased (Supplementary Data S1), implying that the band might be a blend of MspA-D. Noticeably, the expression of *mspB* underwent substantial upregulation, demonstrating an mRNA level increase exceeding threefold in Δ*mstime* when compared to the wild-type *M. smegmatis*. Taken together, the mycomembrane porin MspB and its paralogues showed a greater induction to compensate for the deficiency in Δ*mstime*. Consequently, we postulate that msTiME might potentially possess a nutrient transportation function similar to MspA. 

### 3.3. TiME Benefits Monosaccharide Uptake in M. smegmatis

To investigate the role of msTiME in nutrient transportation, we utilized constitutive expression plasmids integrated into attachment sites to supplement the msTiME level (Supplementary Table S1). The msTiME proteins form tubular complexes spanning the mycobacterial envelope, while msTiME ^G33R-G50R-A91R^ mutant proteins disrupt the ring-shaped structure, impairing pore-forming activity [14]. Therefore, six strains (wild-type, Δ*mstime*, Δ*mstime*::*mstime*, Δ*mstime*::*mstime*^G33R-G50R-A91R^ mutant, Δ*mstime*::*mttime*, and Δ*mstime*::*mttime*^G31R-G48R-A89R^ mutant) were constructed and employed in a saccharide uptake assay.

**Figure 2:**
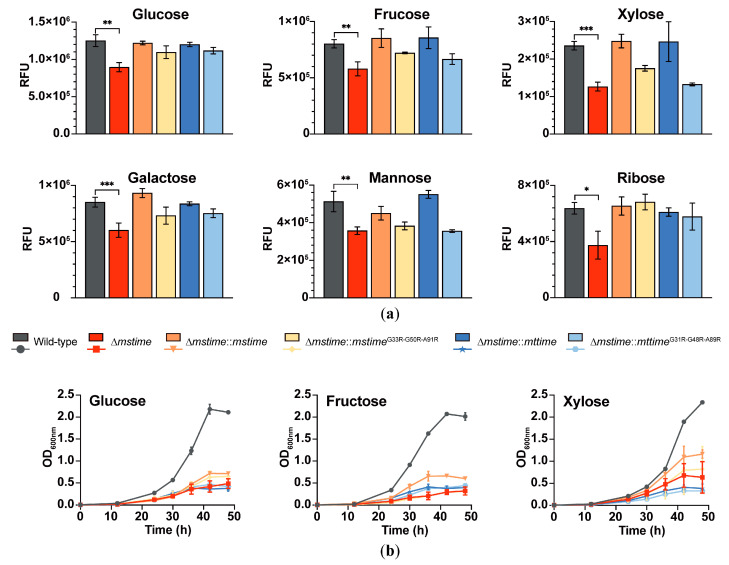
Different monosaccharide uptake assays and growth curves of six *M. smegmatis* strains without detergent Tyloxapol. (**a**) Uptake assay of *M. smegmatis* strains with different monosaccharides. The accumulated fluorescence intensities of different monosaccharides in *M. smegmatis* are depicted. Permeability analyses were independently conducted three times, each in technical triplicate, and the representative experiments are shown as mean ± S.D. (ns, not significant; * indicating *p* < 0.05; ** indicating *p* < 0.01; *** indicating *p* < 0.001 by Student’s paired t-test). RFU denotes relative fluorescence units. (**b**) Growth curves of *M. smegmatis* strain using glucose, fructose, or xylose as the only carbon source. The strains were cultured in 7H9 medium containing 0.2% (*w*/*v*) monosaccharide as the carbon source without 0.02% (*w*/*v*) Tyloxapol. Bacterial growth was assessed by measuring the OD_600nm_ of the bacteria suspension. The results shown represent three independent experiments as mean ± S.D. (n = 3).

Fluorescein-tagged monosaccharides (Supplementary Table S2) served as probes to measure their accumulations in the six strains, displaying varied levels of NBD-tagged monosaccharides uptake through fluorescence intensity detection. Divergent outcomes were evident between the wild-type and Δ*mstime* strains cultured without or with tyloxapol to disrupt the capsule and surface of the cell wall (Figure 2a and Supplementary Figure S3a). In strains with an intact envelope, msTiME facilitated monosaccharide uptake. Notably, Δ*mstime* exhibited approximately a 30% reduction in permeability for glucose, fructose, mannose, galactose, and ribose compared to the wild-type in strains’ coated capsule and an ~50% reduction for xylose uptake (Figure 2a). These findings suggested that the absence of msTiME impeded the monosaccharide uptake in an undamaged envelope. Additionally, constitutive expressing of msTiME rescued the monosaccharide uptake deficiency in the Δ*mstime* strain with a coated capsule. Moreover, the compensatory effect of msTiME and mtTiME was fundamentally similar. It was shown that the permeability coefficient of Δ*mstime* for these monosaccharides had either returned to the wild-type level or surpassed it, contingent upon the expression of TiME and the formation of a ring-shaped structure. These findings substantiate that the ring-shaped TiME complex traversing the mycobacterial outer envelope assists in transporting monosaccharides. 

Conversely, in strains with the surface layer removed, the wild-type strain exhibited lower permeability for glucose, galactose, and xylose compared to Δ*mstime*. Unexpectedly, regardless of whether msTiME, mtTiME, or their variants were reverse expressed, Δ*mstime* strains lacking a surface layer displayed an improved permeability for these monosaccharides compared to the wild-type (Supplementary Figure S3a). This outcome could be attributed to the compromised mycobacterium envelope barrier and the upregulation of certain sugar transporters and porins after *mstime* knocking out, aligning with the aforementioned transcriptome comparison results. Based on these results, we conclude that TiME influences the absorption of monosaccharides at the outer mycobacterial envelope. 

### 3.4. TiME Facilitates Growth of M. smegmatis on Different Monosaccharides

To extensively probe TiME’s role in carbohydrate metabolism, the six M. smegmatis strains were cultivated in the media using monosaccharide as the sole carbon source. However, not all mentioned saccharides could suffice as a carbon source for bacterial proliferation despite being aided by TiME in absorption. 

TiME indeed enhanced the influx of glucose, fructose, and xylose across the outer envelope (Figure 2b). In the wild-type strain with a capsule, growth into the stationary phase using glucose, fructose, or xylose alone exhibited a density of over four times greater than that of the Δ*mstime* strain (Figure 2b), indicating a significant growth impairment in Δ*mstime* with a coated surface layer. The constitutive expression of msTiME partially restored the growth defect observed in Δ*mstime* with a coated capsule (Figure 2b). However, this effectiveness was limited in the *mstime*^G33R-G50R-A91R^ mutant and the homologous TiME from Mtb. Moreover, no significant growth difference was observed among the different strains cultured with detergent to eliminate the capsule and cell wall surface (Supplementary Figure S3b). 

Experimental evidence confirmed that msTiME was necessary for saccharide uptake and normal growth in *M. smegmatis* with thick capsules, supporting the saccharide permeability function of msTiME. Considering that both the msTiME and msTiME^G33R-G50R-A91R^ mutant increased monosaccharide permeability in the outer mycobacterial envelope, while complete ring formation or channel-forming activity was essential for intracellular monosaccharide utilization and metabolism, these results strongly suggest that the tubes formed by TiME mediate nutrient transportation.

### 3.5. TiME Is Required for Transportation of Amino Acids by M. smegmatis

**Figure 3:**
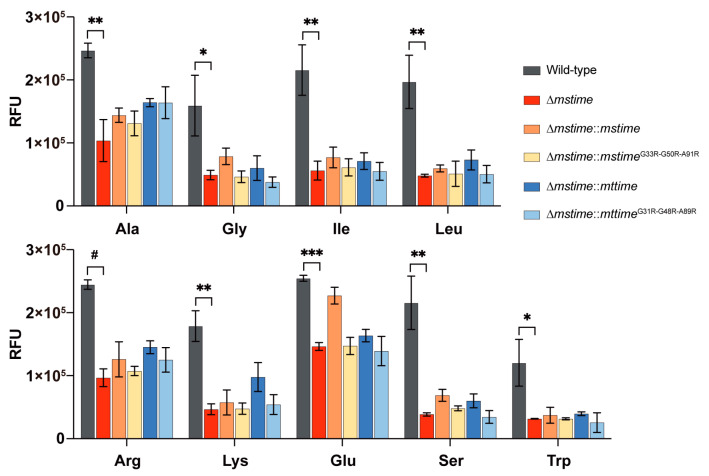
TiME is related to amino acid transportation in *M. smegmatis*. Strains were cultured in the limiting media without Tyloxapol detergent using different amino acids as carbon and nitrogen sources. The assay was conducted independently three times, each with a technical triplicate. The representative experiments are shown as mean ± S.D. (* indicating *p* < 0.05; ** indicating *p* < 0.01; *** indicating *p* < 0.001; ^#^ indicating *p* < 0.0001 by Student’s paired *t*-test). RFU denotes relative fluorescence units.

To explore whether msTiME, akin to MspA, plays a role in transporting other substances, we compared the bacterial activity of the *M. smegmatis* strains cultured in minimal Sauton’s medium supplemented with different amino acids based on our earlier transcriptome finding. Nine amino acids were selected as both the carbon source and nitrogen source, and as anticipated, Δ*mstime* exhibited a growth deficiency in the limiting detergent-free media (Figure 3). When observing the growth of strains with capsules in different amino acids, the density of the wild-type was also significantly higher than that of Δ*mstime*, which showed evident growth retardation. Despite the variations among the strains, the complementary strains, except for Δ*mstime*::*mstime* cultured in the glutamine medium, failed to fully restore the growth levels, similar to the wild-type. Thus, TiME indeed participates in the uptake of amino acids in *M. smegmatis*, requiring other proteins to facilitate amino acid metabolism.

### 3.6. TiME Aids the Resistance of M. smegmatis against Anti-TB Drugs

**Figure 4:**
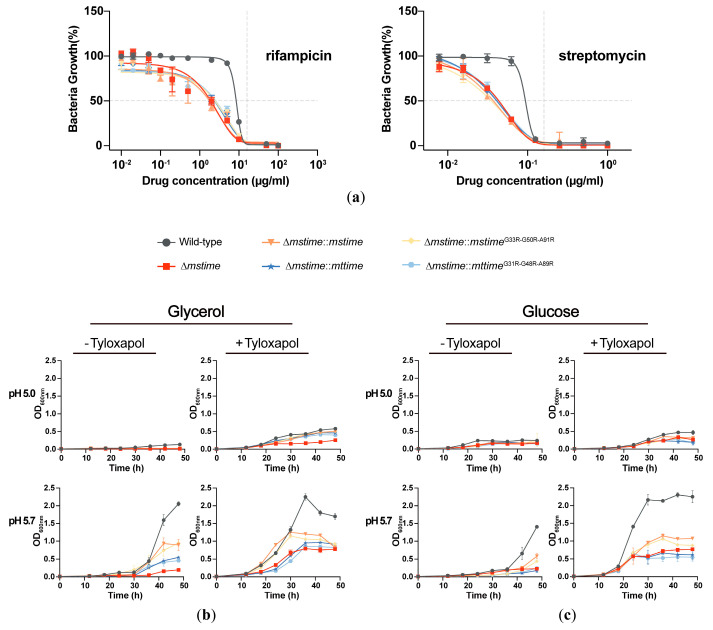
TiME’s role in anti-TB drug susceptibility and acidic pH-dependent growth in *M. smegmatis*. (**a**) Susceptibility of *M. smegmatis* strains to rifampicin and streptomycin in media without Tyloxapol detergent. Data are representative of two independent experiments, both done as technical duplicates, and error bars represent S.D. [in rifampicin: *p* < 0.0001; in streptomycin: *p* = 0.0005; wild-type versus Δ*mstime*; data were analyzed using two-way analysis of variance (ANOVA) of matched values]. (**b**,**c**) Growth of *M. smegmatis* stains in the presence or absence of Tyloxapol using glycerol (**b**) or glucose (**c**) as the sole carbon source. Bacteria were cultured at pH 5.0 and 5.7, respectively. The growth curve was determined by measuring the OD_600_ of bacterial suspension, indicating mean ± S.D. (n = 3).

MspA has previously been shown to enhance the outer membrane permeability of Mtb and M. bovis BCG to antibiotics, as strains expressing mspA exhibit increased sensitivity to antibiotics [17]. To assess the significance of the TiME porins in mycobacterial drug susceptibility, we compared the resistance of wild-type and Δ*mstime* strains to anti-TB drugs. Surprisingly, deletion of the msTiME gene drastically heightened the sensitivity of *M. smegmatis* to rifampicin and streptomycin in media without detergent (Figure 4a). Rifampicin had a modest half-maximal inhibitory concentration (IC_50_) of approximately 1.08 μg/mL for Δ*mstime*, whereas the wild-type strain exhibited a much higher IC_50_ (8.17 μg/mL), despite both strains displaying similar minimum inhibitory concentrations (MICs) of around 16 μg/mL. As for streptomycin, both the wild-type and Δ*mstime* strains had MICs focused around 0.16 μg/mL. However, Δ*mstime* exhibited an approximately two-fold decreased IC_50_ (0.046 μg/mL) compared to the wild-type (0.088 μg/mL). Nevertheless, complementation of Δ*mstime* with either the msTiME or mtTiME genes failed to restore resistance to anti-TB drugs.

### 3.7. TiME Is Essential for Efficient Growth by M. smegmatis at Acidic pH

In tuberculosis pathogenesis, Mtb thrives in environments characterized by an acidic pH and limited carbon sources, often experiencing growth arrest under such conditions [30]. To investigate the potential role of TiME protein in bacterial growth and carbon source utilization at a low pH, *M. smegmatis* wild-type and Δ*mstime* strains were cultured in a defined minimal medium, buffered at pH 5.0 and pH 5.7, utilizing glycerol (Figure 4b) or glucose (Figure 4c) as single carbon sources [31]. Obviously, *M. smegmatis* failed to grow at pH 5.0. At pH 5.7, the Δ*mstime* strain exhibited impaired growth, with the bacterial density remaining lower by threefold compared to the wild-type during the stationary phase. This growth disparity was notably accentuated in strains with an enclosed capsule layer. Remarkably, Δ*mstime* failed to attain optical densities comparable to the wild-type strains after logarithmic growth at pH 5.7, emphasizing the significance of msTiME for efficient growth in *M. smegmatis*. The distinct phenotype of the Δ*mstime* with a capsule layer was particularly pronounced at pH 5.7, underscoring the role of the TiME channel in nutrient uptake and the survival of *M. smegmatis* in acidic environments.

## 4. Discussion

Mycobacteria are classified into two phenotypes based on their growth characteristics: rapid (e.g., *M. smegmatis*) and slow (e.g., *M. tuberculosis*) growers [32]. While porin or channel activity proteins in the outer mycobacterial envelope have been observed, the mechanisms of outer membrane transportation in Mtb remain enigmatic. MspA, lacking a homologous protein in slow-growing mycobacteria, has been identified as the major porin traversing the cell wall of *M. smegmatis*, acting as the primary hydrophilic pathway for nutrient influx [18]. MspB, as the backup porin for MspA, is induced when *mspA* is absent, along with the induction of MspD expression [33]. In our study, deleting *mstime* in *M.smegmatis* also led to a significant upregulation of *mspB* (Figure 1a,d, Supplementary Data S1 and S2). Structurally, TiME is larger than MspA, and TiMEs form a ~50 Å inner diameter complex, allowing for an easier molecular passage compared to MspA, which has an inner diameter of ~10 Å at its narrowest point (Supplementary Figure S1).

In the medium containing detergent Tween80, the Δ*mspA* strain showed a significantly reduced growth rate. The deletion of both *mspA* and *mspC* genes further decelerated the growth rate, resulting in a more than 1.5 times lower bacterial density compared to the wild-type *M.smegmatis* at the stationary phase [33]. The accumulation of glucose by the wild-type strain was nearly twice as high as that by Δ*mspA* after a 15-min incubation [33]. Conversely, Δ*mstime* and the wild-type strain exhibited similar growth rates in the medium with detergent (Supplementary Figure S3). When the outermost envelope of *M.smegmatis* is removed, bacteria can more easily acquire substances from the environment. Despite the deficiency of TiME protein, the upregulated sugar transporters and porins assist *M.smegmatis* in absorbing carbohydrates and transporting them to intracellular metabolism, as demonstrated by the results of monosaccharide absorption and growth experiments (Supplementary Figure S3). In the strains with an intact envelope, the uptake of different monosaccharides was significantly lower, and the growing without detergent led to more clumping cells, highlighting a reduced growth rate and density in Δ*mstime* compared to the wild-type (Figure 2 and Figure 3). These observations suggest a functional similarity between MspA and TiME. 

CpnT (Rv3903c) from Mtb has also been suggested as a channel-forming protein for nutrient uptake via the N-terminal channel domain, but no evidence of subcellular localization has been provided to conclusively prove that CpnT is an outer membrane protein [34,35]. The *cpnT*::*Tn* mutant (*bcg3960c*) of *M. bovis* displayed upregulation of three genes (*bcg0940*, *bcg2411*, *bcg3764c*) predicted to encode outer membrane proteins based on the transcriptome comparison. This mutant grew much better in a medium containing glucose as the sole carbon source compared to the wild-type *M. bovis* [35]. The bcg_3764c gene encoded the conserved protein TiME, suggesting that upregulated TiME might contribute to more efficient glucose utilization. Consistently, our studies represented that an intact TiME channel was required for the permeability and utilization of carbohydrates in *M.smegmatis*. Additionally, the uptake and growth deficiencies observed in the *cpnT* deletion mutant were rescued by the expression of MspA in *M. bovis* [34]. These results further indicate a similar trend between the TiME and MspA genes. 

Furthermore, the expression of MspA in Mtb substantially reduced virulence and enhanced immunogenicity [36]. In like manner, the overexpression of TiME (MAV_0398) in *Mycobacterium avium subsp*. *Hominissuis* resulted in decreased survival after infecting THP-1 cells [37]. Thus, there are similarities between the roles of MspA and TiME in pathogenesis. However, their functions in susceptibility to anti-TB drugs are opposite. MspA porin facilitates an easier entry of antibiotics into the cells, rendering mycobacteria more sensitive to anti-TB drugs [17], while the deletion of TiME increases susceptibility to anti-TB drugs. It is noteworthy that the MICs of the wild-type and Δ*mstime* are nearly the same, whereas there is a notably higher IC_50_ of the wild-type in comparison to Δ*mstime*, which might be attributed to an alternate function of TiME in secretion. Moreover, the complementation of Δ*mstime* failed to counteract the effects of anti-TB drugs, suggesting that the function of TiME might be regulated by other proteins. Despite the highly similar structures of msTiME and mtTiME and their similar effects on monosaccharide absorption, the complementation effect of mtTiME is not as good as msTiME in Δ*mstime* growth. This discrepancy might stem from the fact that the uptake of monosaccharides and amino acids into the cell requires collaboration with other transporters. 

On the other hand, the secretion of the TiME (MAV_0398) protein increased following the stimulation of *M. avium* using an in vitro system that mimicked the phagosomal environment [37]. Our data also showed that *M. smegmatis* expressing TiME proteins exhibited enhanced growth efficiency at acidic pH. These findings indicate that TiME contributes to the adaptive response of mycobacteria under acidic pH conditions. 

**Figure 5:**
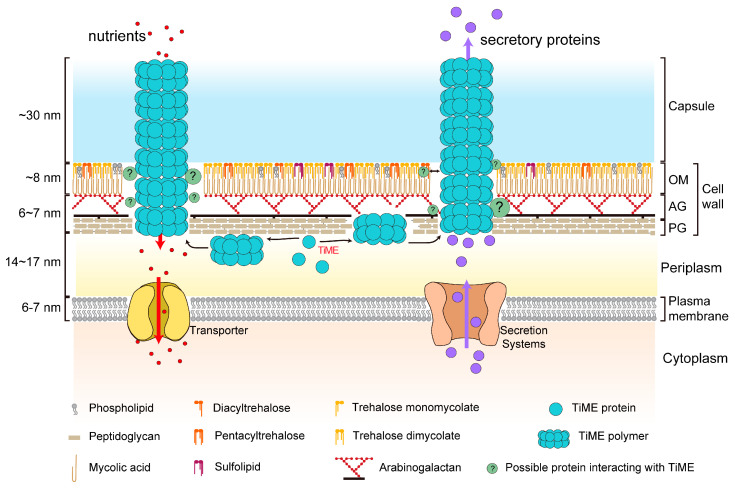
A proposed model for nutrient uptake and protein secretion pathway of TiME in Mycobacterial envelope. TiME proteins self-assemble into rings and stack into double-layered complexes, forming tubular structures traversing the cell wall and capsule. The TiME tube facilitates nutrient uptake (red arrows) from the extracellular environment and transports proteins (purple arrows) from the periplasm to the extracellular space. OM, outer membrane or mycomembrane; AG, arabinogalactan; PG, peptidoglycan; PM, plasma membrane.

Based on previous reports and our aforementioned results, we propose that TiME functions as a bi-directional channel, enabling both nutrient uptake and secretory activities (Figure 5), promoting the thriving of mycobacteria in hostile environments. A typical bi-directional channel in biomembrane is Aquaporin, which facilitates the bi-directional flow of water and small, neutral solutes down an osmotic gradient [38]. However, no bi-directional channel on the mycobacterial outer membrane has been reported so far. Although PPE51 (Rv3136), localized on the outer membrane of Mtb, mediates nutrient transport [20], no corresponding homologous protein in *M.smegmatis* has been identified with a similar function. Given the high conservation of the sequence and three-dimensional structure of TiME in *M. smegmatis* and *M. tuberculosis* [14], as well as the observed role of the TiME secretion channel in the nutrient uptake of *M. bovis* and *M. avium*, it is plausible to extrapolate that TiME could function as a conserved bi-directional channel on the outer envelope of mycobacteria, which would be explored in greater detail on Mtb.

The potential of the TiME channel as a novel target for drug or vaccine development against TB remains an area for further exploration. Additionally, the full-length TiME protein, with its N-terminal helix α0, forms a transmembrane pore in the lipid bilayer that is non-voltage-gated and permits the passage of molecules [14]. This characteristic suggests that TiME could function as a nanopore trap, imitating MspA, to investigate protein activities or to distinguish between different proteins directly [39,40,41]. The larger nanopores formed by TiME may be engineered as sensors for detecting macromolecules or complex assemblies, such as viruses or protein complexes. TiME could also be utilized for nanoparticle characterization or in drug delivery applications.

## 5. Conclusions

This study unveils the dual role of TiME as both a protein secretion channel and a mediator of nutrient transport across the mycobacterial envelope. The absence of TiME in *M. smegmatis* led to a compensatory induction of carbon-hydrate transporters and porins, mitigating the nutrient uptake deficiency. Our data also suggest that TiME plays a pivotal role in facilitating the adaptive responses of *M. smegmatis* to both antibiotics and acidic environments. TiME’s high conservation across mycobacterial species, coupled with its lack of sequence similarity to any human protein, highlights its potential as a promising drug target against tuberculosis and other mycobacteria-induced diseases. The novel structure and function of TiME could serve as a new nanopore trap for various applications.

## Data Availability

The data are available in the article or its Supplementary Materials.

## Supplementary Materials

The following supporting information can be downloaded at: https://www.mdpi.com/article/10.3390/microorganisms12091827/s1, Figure S1: Structural features of MspA and TiME; Figure S2: GO enrichment and COG enrichment analyses of annotated DEGs; Figure S3: Different monosaccharide uptake assays and growth curves of six *M. smegmatis* strains with Tyloxapol detergent; Table S1: Strains and plasmids used in this study; Table S2: NBD-tagged monosaccharides; Table S3: Broth mediums for culturing strains in different assays; Table S4: Primers used in this study. Data S1: DEGs between WT and *mstime* knock-out strains; Data S2: mass spectrometry result of SDS-PAGE band. References [14,42,43,44] are cited in the Supplementary Materials.
