# Supplementary material for: A Novel Bi-Directional Channel for Nutrient Uptake across Mycobacterial Outer Envelope
Source: Microorganisms. 2024 Sep 4;12(9):1827. doi: 10.3390/microorganisms12091827 (PMC11434571; doi:10.3390/microorganisms12091827)
Supplement: Supplementary file 1 [file microorganisms-12-01827-s001.zip › microorganisms-3112793-supplementary-V3/Supplementary materials.pdf]

Supplementary materials

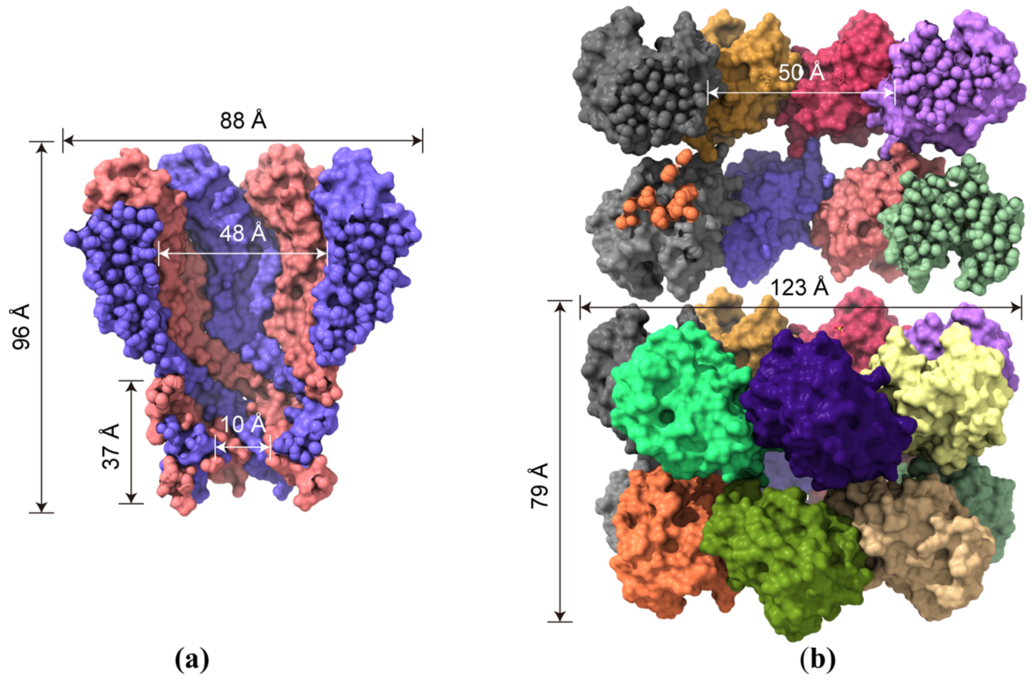

Supplementary Figure S1. Structural features of MspA and TiME. (**a**) The goblet-like structure of MspA from *Mycobacterium smegmatis* (PDB code 1UUN) is displayed in a cross-sectional view. (**b**) Tube structural model of TiME from *Mycobacterium tuberculosis* (PDB code 7CU8). The upper two rings are shown in a cross-sectional view.

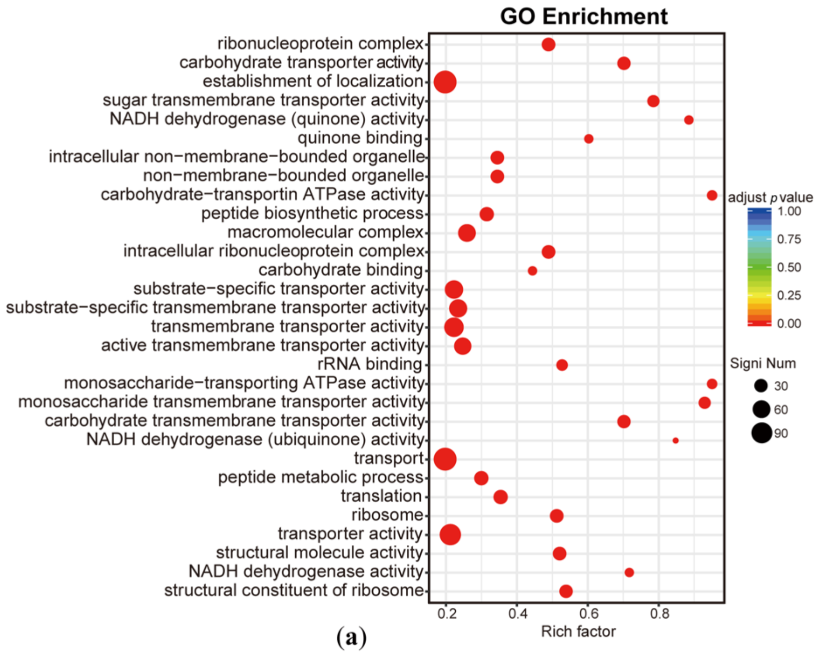
GO Enrichment
ribonucleoprotein complex
carbohydrate transporter activity
establishment of localization
sugar transmembrane transporter activity
NADH dehydrogenase (quinone) activity
quinone binding
intracellular non−membrane−bounded organelle
non−membrane−bounded organelle
carbohydrate−transportin ATPase activity
peptide biosynthetic process
macromolecular complex
intracellular ribonucleoprotein complex
carbohydrate binding
substrate−specific transporter activity
substrate−specific transmembrane transporter activity
transmembrane transporter activity
active transmembrane transporter activity
rRNA binding
monosaccharide−transporting ATPase activity
monosaccharide transmembrane transporter activity
carbohydrate transmembrane transporter activity
NADH dehydrogenase (ubiquinone) activity
transport
peptide metabolic process
translation
ribosome
transporter activity
structural molecule activity
NADH dehydrogenase activity
structural constituent of ribosome
0.2
0.4
0.6
0.8
Rich factor
adjust p value
1.00
0.75
0.50
0.25
0.00
Signi Num
30
60
90

(a)

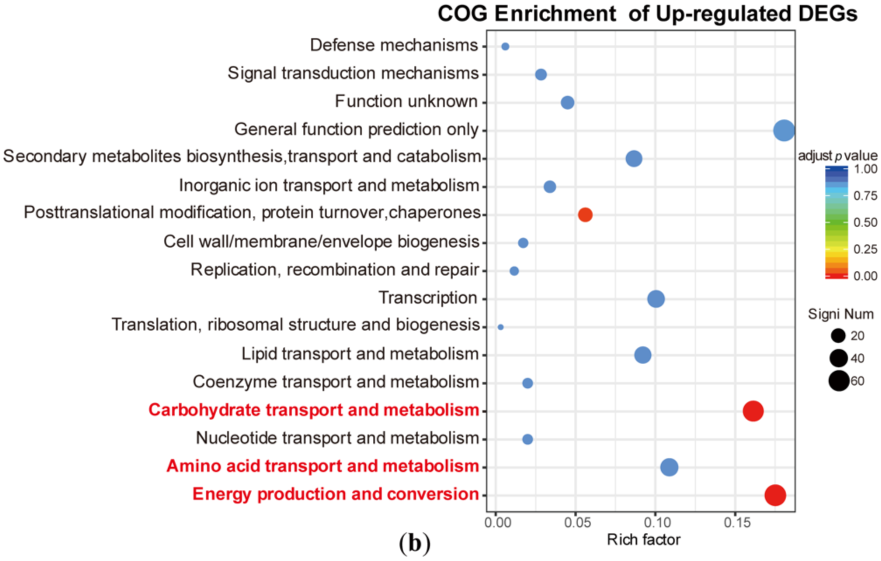
COG Enrichment of Up-regulated DEGs
Defense mechanisms
Signal transduction mechanisms
Function unknown
General function prediction only
Secondary metabolites biosynthesis,transport and catabolism
Inorganic ion transport and metabolism
Posttranslational modification, protein turnover,chaperones
Cell wall/membrane/envelope biogenesis
Replication, recombination and repair
Transcription
Translation, ribosomal structure and biogenesis
Lipid transport and metabolism
Coenzyme transport and metabolism
Carbohydrate transport and metabolism
Nucleotide transport and metabolism
Amino acid transport and metabolism
Energy production and conversion
0.00
0.05
0.10
0.15
Rich factor
adjust p value
1.00
0.75
0.50
0.25
0.00
Signi Num
20
40
60

(b)

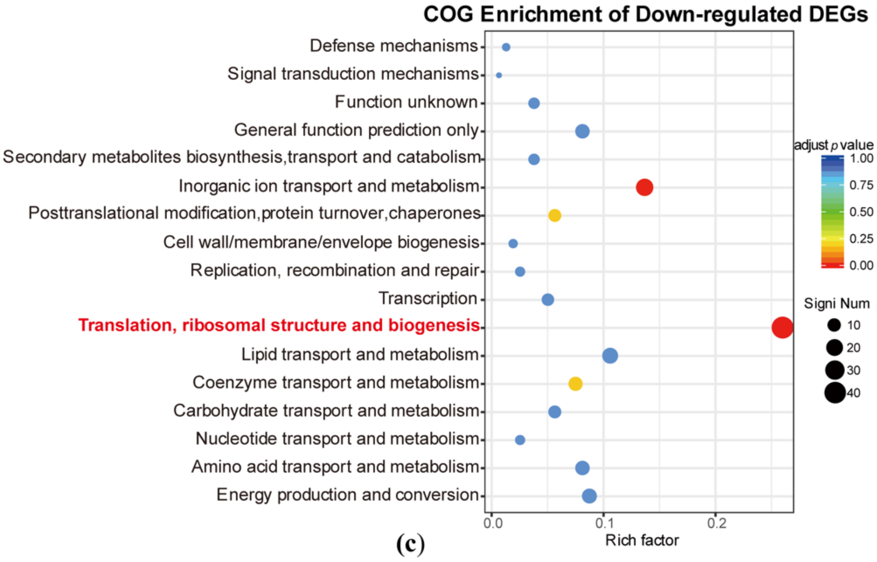
COG Enrichment of Down-regulated DEGs
Defense mechanisms
Signal transduction mechanisms
Function unknown
General function prediction only
Secondary metabolites biosynthesis,transport and catabolism
Inorganic ion transport and metabolism
Posttranslational modification,protein turnover,chaperones
Cell wall/membrane/envelope biogenesis
Replication, recombination and repair
Transcription
Translation, ribosomal structure and biogenesis
Lipid transport and metabolism
Coenzyme transport and metabolism
Carbohydrate transport and metabolism
Nucleotide transport and metabolism
Amino acid transport and metabolism
Energy production and conversion
0.0
0.1
0.2
Rich factor
adjust p value
1.00
0.75
0.50
0.25
0.00
Signi Num
10
20
30
40

(c)

Supplementary Figure S2. GO enrichment (**a**) and COG enrichment (**b** and **c**) analysis of annotated DEGs. The functions enriched among the DEGs were categorized, presenting the top 30 (or 17) enriched functions. 'Rich factor' refers to the ratio of DEGs to all genes within a specific functional category. Dot size indicates the number of DEGs in the pathway, while dot color reflects the adjusted *p* value. 'Signi Num' represents the number of significant genes.

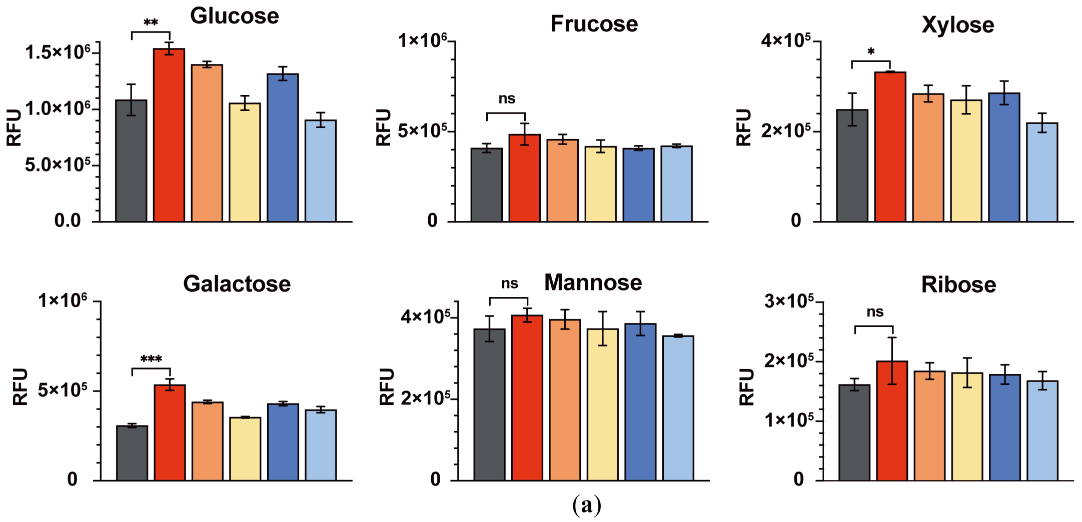

(**a**)

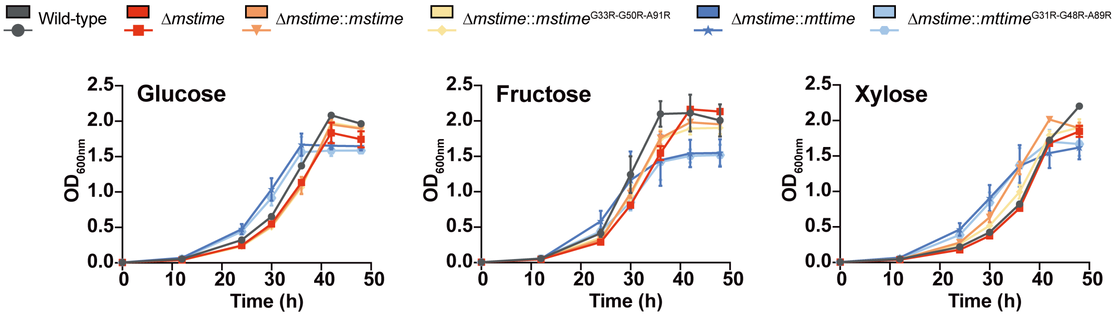

(**b**)

Supplementary Figure S3. Different monosaccharides uptake assay and growth curve of six *M. smegmatis* strains with Tyloxapol detergent. (**a**) Uptake assay of *M. smegmatis* mc²155 strains with different monosaccharides. Permeability analyses were performed independently three times, each in technical triplicate and representative experiments are shown as mean±S.D. (ns, not significant; *indicating $p<0.05$; ** indicating $p<0.01$; *** indicating $p<0.001$ by Student's paired t-test). (**b**) Growth curves of *M. smegmatis* mc²155 strains with glucose, fructose, or xylose as only carbon source. The growth was determined by measuring the OD_{600nm} of the bacteria suspension. Results shown are representative of three independent experiments as mean±S.D. (n=3).

Supplementary Table S1. Strains and plasmids used in this study.

Strain/Plasmid	Description	Source or Ref.
M. smegmatis mc^{2}155	Wild-type *M. smegmatis* mc^{2}155 strain	[1]
Δ*mstime M. smegmatis* mc^{2}155	*mstime* (*msmeg_6251*) gene knock-out *M. smegmatis* mc^{2}155 strain	[2]
Δmstime::mstime M. smegmatis mc^{2}155	*msmeg_6251* gene knock-out *M. smegmatis* mc^{2}155 strain with complementary msTiME	[2]
Δmstime::mstime $^{G33R\text{-}G50R\text{-}A91R}$ mutant *M. smegmatis* mc^{2}155	*msmeg_6251* gene knock-out *M. smegmatis* mc^{2}155 strain with complementary msTiME$^{G33R\text{-}G50R\text{-}A91R}$ mutant	[2]
Δmstime::mttime M. smegmatis mc^{2}155	*msmeg_6251* gene knock-out *M. smegmatis* mc^{2}155 strain with complementary mtTiME	This study
Δmstime::mttime $^{G31R\text{-}G48R\text{-}A89R}$ mutant *M. smegmatis* mc^{2}155	*msmeg_6251* gene knock-out *M. smegmatis* mc^{2}155 strain with complementary mtTiME$^{G31R\text{-}G48R\text{-}A89R}$ mutant	This study
pJV53-GFP	*M. smegmatis* mc^{2}155 knock-out strain construction vector	[2]
PUC-Hyg-P1-P2[a]	Δ*msmeg_6251 M. smegmatis* mc^{2}155 strain construction plasmid	[2]
pMV361D[b]	*M. smegmatis* mc^{2}155 strain constitutive expression vector	[2]
pMV361D-*mstime*	msTiME constitutive expression plasmid	[2]
pMV361D-*mstime*-G33R-G50R-A91R	msTiME$^{G33R\text{-}G50R\text{-}A91R}$ constitutive expression plasmid	[2]
pMV361D-*mttime*	mtTiME constitutive expression plasmid	This study
pMV361D-*mstime*-G31R-G48R-A89R	mtTiME$^{G31R\text{-}G48R\text{-}A89R}$ constitutive expression plasmid	This study

[a] Hyg^r is hygromycin resistance. (50 μg/mL for *M. smegmatis*)

[b]Kan^r is kanamycin resistance. (50 μg/mL for *M. smegmatis*)

Supplementary Table S2. Primers used in this study.

Name	Sequence (5' to 3' direction)	Use
pMV361-ATG-F	GGAGGAATCACTTCGCAGCCAAGACAATTGCG	Mutagenesis
pMV361-ATG-R	CGCAATTGTCTTGGCTGCGAAGTGATTCCTCC	Mutagenesis
pMV361D-mstime-F	GACGAATTCATGATCGAGCCCATGCGTG	Constitutive expression
pMV361D-mstime-R	CCCAAGCTTTTATTGCCCCAGGGTGTTCTGC	Constitutive expression
pMV361D-mttime-F	GACGAATTCATGCGAATCGCCGCCGCGG	Constitutive expression
pMV361D-mttime-R	CCCAAGCTTTCAGCCCAGTGTGTTCTGCATTGCCC	Constitutive expression

Supplementary Table S3. Broth mediums for culturing strains in different assays.

Assays	Ingredients		Medium name
Culturing	0.47% Middlebrook 7H9 dehydrated broth (BD Difco^{TM}); 0.2% (v/v) glycerol		7H9 broth A
Growth on different monosaccharides	0.47% Middlebrook 7H9 dehydrated broth (BD Difco^{TM}); 0.2% (w/v) monosaccharides		7H9 broth B
Growth on different amino acids	K_2HPO_4	2.9 mM	minimal Sauton's medium [3]
	$MgSO_4$	2 mM	
	sodium citrate	9.5 mM	
	ferric ammonium citrate	0.2 µM	
	Amino acid	10mM	
	pH	7.0	
Growth at acid pH	KH_2PO_4	1 g/L	minimal medium [4]
	$Na_2HPO4 \cdot 12H_2O$	2.5 g/L	
	$(NH4)_2SO_4$	0.5 g/L	
	asparagines	0.15 g/L	
	$MgSO_4$	10 mg/L	
	ferric ammonium citrate	50 mg/L	
	$ZnSO_4 \cdot 7H_2O$	0.1 mg/L	
	$CaCl_2$	0.5 mg/L	
	Glycerol or Glucose	0.2% (v/v) or 0.2% (w/v)	
	HCl: 5.0	pH*	
	MES: 5.7	pH*	

* pH is adjusted by adding HCl or MES to the defined minimal medium before autoclave sterilization.
Tyloxapol detergent is used to peel off mycobacterial capsule and surface of cell wall.

Supplementary Table S4. NBD-tagged monosaccharides.

Monosaccharide	Formal Name	Source
NBD-Glucose	2-deoxy-2-[(7-nitro-2,1,3-benzoxadiazol-4-yl)amino]-D-glucose	Cayman
NBD-Fructose	1-deoxy-1-[(7-nitro-2,1,3-benzoxadiazol-4-yl)amino]-D-Fructose	Heowns
NBD-Galactose	2-deoxy-2-[(7-nitro-2,1,3-benzoxadiazol-4-yl)amino]-D-Galactose	Heowns
NBD-mannose	2-deoxy-2-[(7-nitro-2,1,3-benzoxadiazol-4-yl)amino]-D-Galactose	Heowns
NBD-Ribose	1-deoxy-1-[(7-nitro-2,1,3-benzoxadiazol-4-yl)amino]-D-Ribose	BG Biotech
NBD-Xylose	1-deoxy-1-[(7-nitro-2,1,3-benzoxadiazol-4-yl)amino]-D-Xylose	BG Biotech

References

1. Mohan, A.; Padiadpu, J.; Baloni, P.; Chandra, N. Complete Genome Sequences of a Mycobacterium smegmatis Laboratory Strain (MC2 155) and Isoniazid-Resistant (4XR1/R2) Mutant Strains. Genome Announc 2015, 3, doi:10.1128/genomeA.01520-14.
2. Cai, X.; Liu, L.; Qiu, C.; Wen, C.; He, Y.; Cui, Y.; Li, S.; Zhang, X.; Zhang, L.; Tian, C.; et al. Identification and architecture of a putative secretion tube across mycobacterial outer envelope. Science Advances 2021, 7, eabg5656, doi:10.1126/sciadv.abg5656.
3. Rieck, B.; Degiacomi, G.; Zimmermann, M.; Cascioferro, A.; Boldrin, F.; Lazar-Adler, N.R.; Bottrill, A.R.; le Chevalier, F.; Frigui, W.; Bellinzoni, M.; et al. PknG senses amino acid availability to control metabolism and virulence of Mycobacterium tuberculosis. PLoS Pathog 2017, 13, e1006399, doi:10.1371/journal.ppat.1006399.
4. Baker, J.J.; Johnson, B.K.; Abramovitch, R.B. Slow growth of Mycobacterium tuberculosis at acidic pH is regulated by phoPR and host-associated carbon sources. Mol Microbiol 2014, 94, 56-69, doi:10.1111/mmi.12688.
